# Theoretical analysis and simulation calculation of hydrodynamic pressure pulsation effect and flow induced vibration response of radial gate structure

**DOI:** 10.1038/s41598-022-26470-x

**Published:** 2022-12-19

**Authors:** Bozhi Zhang, Xiaochuan Jing

**Affiliations:** China Academy of Aerospace Systems Science and Engineering, Beijing, 100035 China

**Keywords:** Energy science and technology, Engineering, Civil engineering

## Abstract

This work aims to explore the characteristics of stochastic fluctuant water pressure acted on the surface of large radial gate, and to investigate the flow-induced vibration response of the whole radial gate structure. The finite element calculation model structure of the radial gate is established by taking a large-scale radial gate as prototype to discuss the hydrodynamic pressure acting on the gate leaf with different opening, analyze the dynamic pressure time curves, and achieve the flow-induced vibration response by deeming hydrodynamic pressure as dynamic load. When taking 10% opening of the radial gate, the results indicate that the hydrodynamic pressure distributed on the arc surface of the radial gate changes with the flow conditions, with the maximum pressure occurred at the center of the lower edge of the gate leaf. One point in the time history curve of fluctuating water pressure can be taken as the dynamic load for the flow-induced vibration analysis. The flow-induced vibration responses at the monitoring point of the radial gate structure show periodic changes in the X, Y, and Z directions. The finite element simulation results agree well with the theoretical calculation results, so reference can be provided for the hydrodynamic pressure testing and the flow-induced vibration response calculations.

## Introduction

Hydraulic gate is the light water retaining structure used most in water conservancy construction projects, so its safety plays a vital role in the whole water conservancy project^1,2^. Among them, radial gate is widely used in various hydraulic structures, for the features of light weight, small opening and closing force, and no gate slot at the bottom edge^3^.

During the opening and closing process or the local opening of radial gate, the gate leaf is under the fluctuating pressure of water flow, thus causing the vibration of the whole gate structure^4,5^. For the calculation of vibration response of radial gate induced by fluctuating water pressure and flow, scholars at home and abroad have conducted numerous theoretical and experimental researches^6^. Liang Chao et al. investigated the gate vibration induced by high dam discharge, found that the gate vibration had the characteristics of energy concentration and frequency stability by adopting the prototype observation method, designed the gate vibration reduction method based on the robust control effect, and discovered that this method had a good effect on the gate vibration reduction after conducting experimental researches^7^. Wang Yanzhao et al. fitted the kinetic energy correction coefficient in the theoretical formula by considering the model test results of three radial gates, and obtained the theoretical method for calculating radial gate panel dynamic water pressure, on the basis of the characteristics of radial gate panel water pressure, and by considering the influence of hinge position and rotating arm radius on panel water pressure^8^. Li Xiaochao et al. analyzed the data of the fluctuating water pressure at a large number of measuring points on the gate panel systematically, and obtained the distribution law of the amplitude and frequency of the fluctuating water pressure on the gate panel, as well as the influence of the gate opening and the upstream and downstream water levels on the fluctuating pressure^9^. Shen Chunying et al. gave research on the fluid structure coupling problem of flat gate, analyzed the data collected by the test instrument, and discovered that the flow-induced dynamic response of the gate was closely related to the flow direction, and the amplitude at the bottom of the gate was closely related to the opening^10^. Zheng Shengyi et al. conducted theoretical research on the fluctuating water pressure on the arc surface of the gate under small opening, calculated the dynamic water pressure on the arc surface theoretically with the flow and superposition principle, as well as the dynamic water force on the gate panel^11^. Guo Guizhen et al. gave experimental research on the structural vibration caused by flow coefficient and fluid inertia, with the focus on the impact of Scruton number on gate stability, and it was discovered after comparison that the experimental results were basically consistent with the gate stability index^12^. Seyed Aghazadeh Banafshe et al. explored the fluid structure coupling mechanism of gate cylinder structure, and discussed the vibration frequency and amplitude after fluid structure coupling^13^. Results of the researches above provide a theoretical basis for the analysis of gate fluctuating pressure and vibration response. However, the theoretical analysis method of fluctuating water pressure needs a large amount of calculation, and it is difficult to obtain the overall distribution of gate leaf pulsating water pressure. Moreover, the field measurement is often limited by the complex structure of gate and the difficult arrangement of measuring points in service environment^14–16^.

Considering the vibration of gate opening induced by fluctuating water pressure and flow, numerous theoretical analysis, simulations, and experimental researches are still needed^17,18^. This paper establishes the finite element model with coupling of fluid field and solid field of radial gate structure based on the engineering background, adopts the method of combining finite element simulation with field measurement, and investigates the distribution of fluctuating water pressure on radial gate leaf and the vibration response of the whole gate structure caused by flow excitation.

## Theoretical background

### Flow-induced vibration formula of radial gate structure

According to the vibration theory and motion formula of solid structure, the whole structure composed of radial gate leaf structure, arm structure, and connecting members can be regarded as a structural vibration system with multi degrees of freedom, with the dynamic formula expressed as follows:

1 $${\mathbf{M}}\ddot{u}{ + }{\mathbf{C}}\dot{u}{ + }{\mathbf{K}}u{ = }{\mathbf{F}}p{(}t{)}$$

where **M** represents the overall mass matrix of gate structure; **C** represents the damping matrix of the gate structure; **K** represents the stiffness matrix of the gate structure; $\ddot{u}$, $\dot{u}$, and *u* represent acceleration vector, velocity vector, and displacement vector of the gate structure, respectively; and **F**_***p***_**(t)** refers to the dynamic action of the gate structure caused by water flow, mainly including the hydrodynamic pressure caused by gate vibration and the fluctuating water pressure caused by the water flow when impacting the static gate.

The vibration of radial gate used under the action of water flow is a random process with the fluid structure coupling effect considered. In this paper, the water disturbed by the radial gate is regarded as an additional mass, and its influence on the overall vibration response of the radial gate structure is considered with the finite element method. Simultaneously, the fluctuating water pressure is mainly obtained by conducting model structure test. By carrying out numerous fluctuating water pressure tests on gate model structure, scholars at home and abroad give the time history of fluctuating water pressure. Based on the existing fluctuating water pressure test results, and by considering the differences of the radial gate geometric parameters, material properties, structural dimensions, head height, gate opening, and other factors, the typical time history of fluctuating water pressure is selected as the dynamic load in this paper, and input to the finite element model structure, with the purpose of analyzing the flow-induced vibration response.

### Hydrodynamic pressure caused by the gate vibration

**Figure 1:**
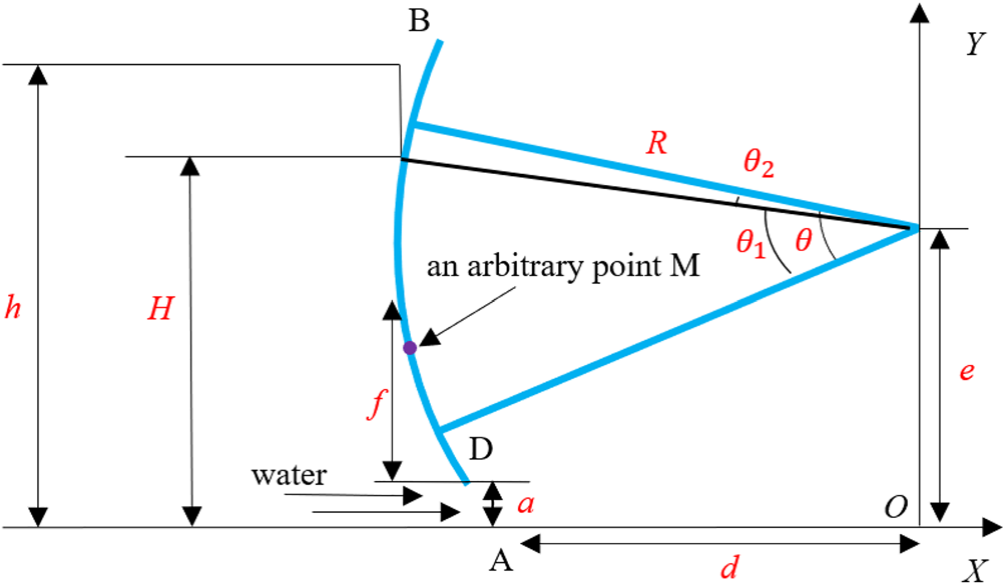
Theoretical model of hydrodynamic pressure of radial gate.

Generally, when not considering the added mass of fluid, the dynamic pressure at a certain point can be calculated with the theoretical formula. The hydrodynamic pressure on leaf structure of radial gate is mainly affected by the size of the water area in front of the gate. According to the hydraulic potential flow theory^19,20^, the hydrodynamic pressure on the leaf of radial gate can be expressed as follows:

2 $${\text{P}}_{{\text{M}}} { = }\beta [h - f - (h - a)k1ck2c/k1k2]$$

where P_M_ represents the hydrodynamic pressure of radial gate panel; *β* represents the bulk density of reservoir water body; *h* represents the water depth in the upstream of the gate; *a* represents the local opening height of the gate; *f* represents the distance from the resultant force center of arc surface to the bottom edge of gate leaf; the specific values of *k*_1*c*_*, k*_2*c*_*, k*_1_*, and k*_2_ can be found in the chart provided in the hydraulic design manual^20^. The theoretical model of hydrodynamic pressure is shown in Fig. 1.

Assuming that M is an arbitrary point on the arc surface of the radial gate leaf, and its coordinate is (*Rcosθ, Rsinθ* + *e*), the flow velocity on the arc surface at point M shall be as below:

3 $$u_{{\text{M}}} = \frac{q}{\pi }\frac{d}{{\sqrt {(x_{{\text{M}}}^{2} - y_{{\text{M}}}^{2} - d^{2} ) + 4x_{{\text{M}}}^{2} y_{{\text{M}}}^{2} } }}$$

When considering points B and M and the Bernoulli formula, the formula below can be obtained.

4 $$y_{{\text{M}}} + \frac{{p_{{\text{M}}} }}{\gamma } + \frac{{u_{{_{{\text{M}}} }}^{2} }}{2g} = H + \frac{{u_{{\text{B}}}^{2} }}{2g} = H_{{0}}$$

From formula (4), the formula of *P*_M_ can be inferred as follows:

5 $$p_{{\text{M}}} = \gamma (H_{{0}} - y_{{\text{M}}} - \frac{{u_{{\text{B}}}^{2} }}{2g})$$

where *P*_M_ represents the hydrodynamic pressure at point M on the arc surface of the gate; *r* represents the unit weight of water; *g* refers to the gravitational acceleration; *H*_0_ value can be determined according to the structural form and opening of radial gate; *y*_M_ and *u*_M_ refer to functions of the variable $\theta$. To solve the hydrodynamic pressure *P* at a certain opening of the gate, it is only necessary to integrate the hydrodynamic pressure strength *P*_M_ along the arc surface, and then multiply it by the width *B*_s_ of the arc surface gate leaf structure.

6 $$P = B_{S} \int_{{\theta {1}}}^{\theta L} {p_{{\text{M}}} } d\theta$$

It can be obtained from the theory of hydrodynamic pressure calculation that the hydrodynamic pressure can be solved by theoretical formula, provided that the position of a certain point of radial gate is known. However, in the practical engineering design and application, it is often necessary to master the distribution of fluctuating water pressure on the whole arc surface with different opening, and the calculation is difficult and even cannot be realized when adopting the theoretical formula. Moreover, as some parts are affected during the operation of the gate, it is hard to fully arrange the measuring points during the field measurement. Thus, the finite element program is used in this paper, to establish the model structure of the radial gate in the fluid field.

When considering the hydrodynamic pressure influence on the overall vibration response of the gate, the water body disturbed by the gate vibration can be deemed as an additional mass in the finite element calculation. When considering the gate possessing flow excitation with multi degrees of freedom, the additional mass dynamics formula can be expressed as below:

7 $$({\mathbf{M}} + {\mathbf{M}}_{A} )\ddot{u} + ({\mathbf{C}} + {\mathbf{C}}_{A} )\dot{u} + ({\mathbf{K}} + {\mathbf{K}}_{A} )u = F^{\prime}p{(}t{)}$$

where **M**_**A**_, **C**_**A**_, and **K**_**A**_ are additional mass matrix, additional damping matrix, and additional stiffness matrix of the water disturbance caused by gate vibration, respectively, and ***F’***_***p***_***(t)*** is the fluctuating water pressure.

### Time history of fluctuating water pressure

In this paper, the time history of fluctuating water pressure **F**_***p***_**(t)** is selected according to the operation of radial gate. The normal pool level is 85.0 m, the maximum dam height is 145.0 m, the width and height of gate leaf of hydraulic radial gate are 8.0 m and 16.3 m, respectively, the radius of curvature of radial gate is 24.0 m, and the gate opening and closing angle is 41.7^°^. According to the principle that the working parameters and structural characteristic parameters of the gate are similar, five groups of time histories of improved fluctuating hydrodynamic pressure are given.

**Figure 2:**
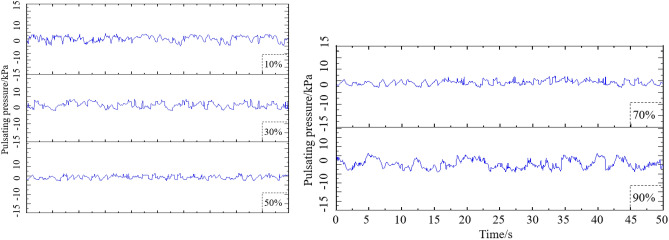
Time history of selected typical fluctuating hydrodynamic pressure.

The existing research results indicate that the variation of fluctuating hydrodynamic dominant frequency of each measuring point shows no obvious fixed law with the change of gate opening under different working conditions, and it varies with the different working conditions and measuring points. In this paper, the finite element model structures with openings of 10%, 30%, 50%, 70%, and 90% are established according to the five curves corresponding to Fig. 2, and the flow-induced vibration response of radial gate structure is calculated and analyzed.

## Simulation and experimental research

### Prototype structure of radial gate and finite element calculation model

**Figure 3:**
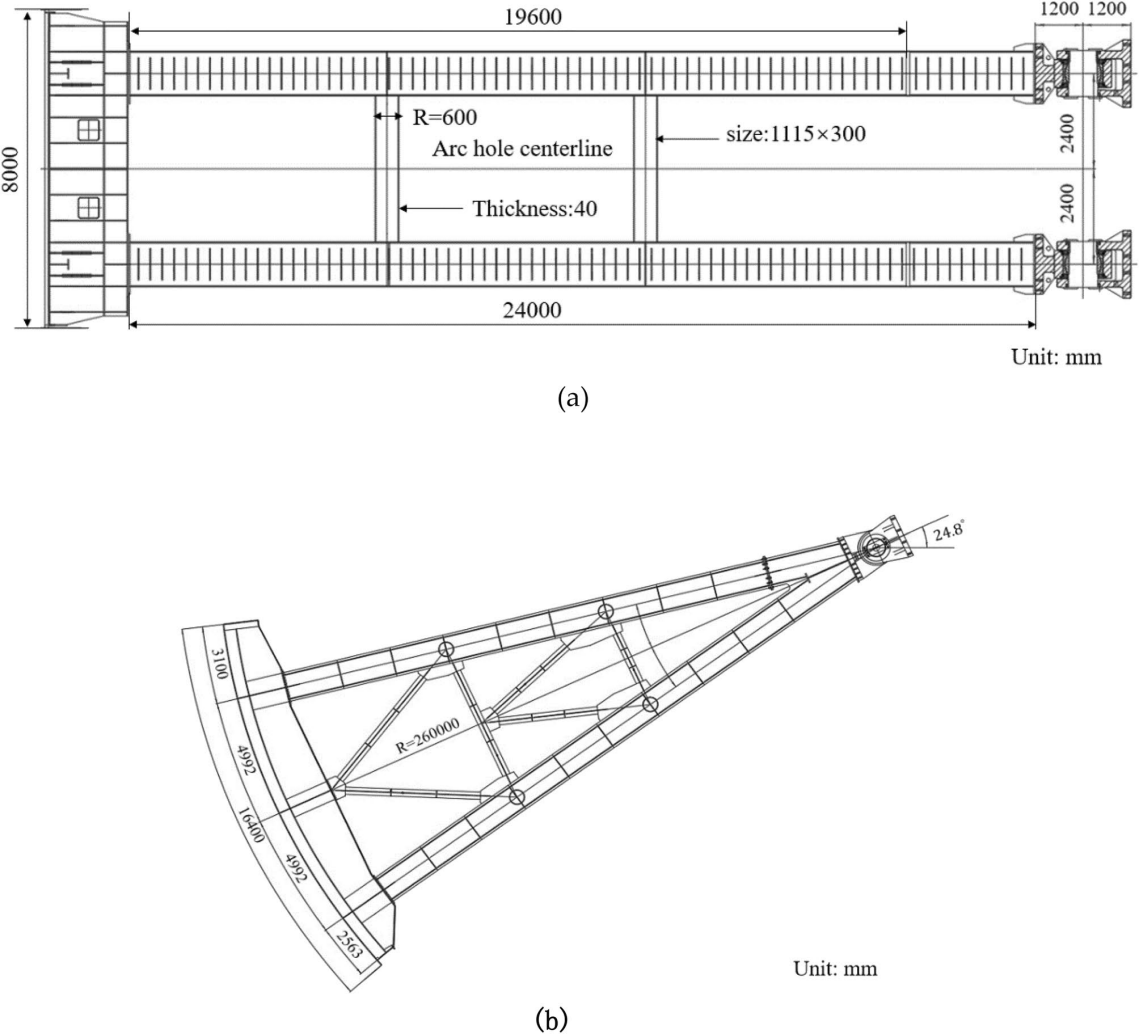
Structural design drawing of radial gate. (**a**) Top view of radial gate, (**b**) Gate structure A-A upstream structural drawing.

In this paper, the radial gate used in an actual project is taken as the prototype structure, as shown in Fig. 3. The full-size finite element calculation model structure of the radial gate is established, and the fluctuating water pressure is applied to the model structure according to the dynamic load, with the purpose of calculating and analyzing the dynamic response of the gate under flow excitation.

The gate leaf structure with radian of 35^°^, arc length of 16,400 mm, radius of 26,000 mm, and width of 8000 mm is established. The gate leaf structure is hollowed out by cutting, stretching, and shell pulling. The cross-sectional dimension of the upper and lower support arms of the radial gate is 1100 mm × 1000 mm, and the connection of the upper and lower arms adopts cold connection. The length of the upper arm is 19,600 mm, that of the lower arm is 24,000 mm, and the arm structure is hollowed out by cutting. The cross beam and secondary beam between the support arms are made of circular steel pipes, with a diameter of 600 mm and a thickness of 40 mm. The hollow rectangular steel member, with dimension of 1115 mm × 300 mm and thickness of 20 mm, is used as the secondary beam structure of the arm structure. Finally, the hinged supports of the gate structure are established, and the dimension of upper support is 1900 mm × 1500 mm, and that of the support is 1800 mm × 1700 mm. All structural members are made of Q345B steel.

**Figure 4:**
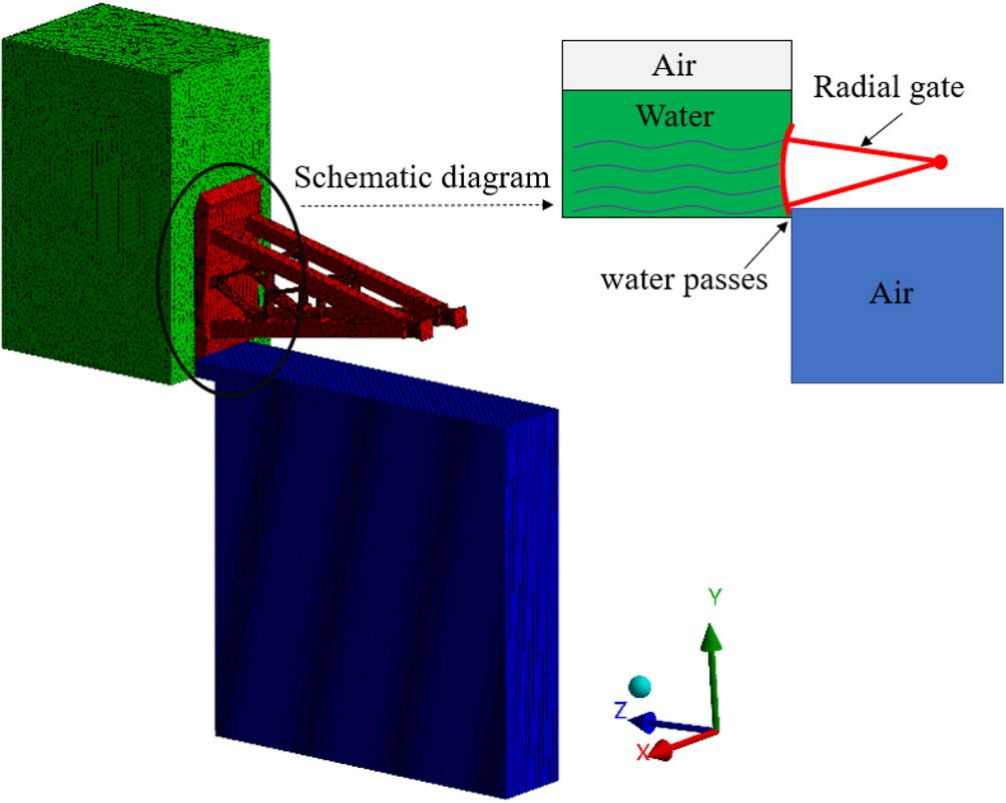
Structure of finite element calculation model for radial gate.

To investigate the hydrodynamic pressure caused by the water disturbance for the radial gate vibration, the area, 20 m in front of the radial gate, 30 m in height, and 15 m in width, is selected as the hydrodynamic pressure water area of the radial retaining surface. The model is imported into the Fluent module in the Ansys Workbench, for the calculation and analysis of fluid field. Then, the model is meshed, with the local area of the model meshed as well, to ensure the correctness of the calculation results of the fluid structure coupling simulation of the radial gate. The wall in the water area is defined as Wall-1 (excepting the top surface), and the junction of the water area and the radial gate is defined as outlet. The wall in the air area at the bottom is defined as Wall-2 (excepting the top surface), and the junction of the air area and the radial gate is defined as inlet. The coupled explicit method is used in calculation and problem solution, and the 50 s solution time is set as 0.1 s/step. Next, the model is initialized, and the displacement and acceleration of the model are calculated and solved. The finite element calculation model of radial gate is shown in Fig. 4.

As shown in Fig. 4, the upstream and downstream water basins with gate structure are set in the finite element model. With the finite element model in Fig. 4, the hydrodynamic pressure caused by water disturbance can be calculated. Simultaneously, by inputting the fluctuating water pressure shown in Fig. 2 in the model, the flow-induced vibration response under the coupling action of gate and water flow can be calculated.

In this paper, the finite element calculation model structure of radial gate is established in the fluid field, with the flow conditions and gate opening conditions set simultaneously. Moreover, the hydrodynamic pressures under different gate opening conditions are simulated and calculated by taking the flow as the object, and the flow-induced vibration responses under the action of hydrodynamic pressure and fluctuating water pressure are simulated and calculated by taking the gate as the research object. Besides, five finite element calculation model structures with different opening heights of 10%, 50%, 70%, 80%, and 90% are calculated according to the working condition, and the vibration response induced by the hydrodynamic pressure and flow and caused by the gate vibration disturbing water body are analyzed.

### Hydrodynamic pressure of whole gate leaf

The hydrodynamic pressure at a certain point of radial gate leaf is usually calculated with the theoretical calculation formula as shown in 2.1. However, in this paper, the flow parameters are set in the finite element model structure as shown in Fig. 4, the standard k-e turbulence model method is selected to simulate the flow effect of water flowing the radial gate leaf, and the flow field material is defined as water liquid from the materials option. In the simulation, when considering that the water flow in the upstream water area may pass through the lower edge of the radial gate structure under the action of gravity during flood discharge, the direction and magnitude of gravity acceleration (− 9.81 m/s^2^) shall be set for calculation. The residual convergence standard for the hydrodynamic pressure in flow field can be determined according to the results obtained from monitors during problem setting. After completing the setting, the hydrodynamic pressure on the radial gate leaf is calculated, and the overall distribution of hydrodynamic pressure is given.

**Figure 5:**
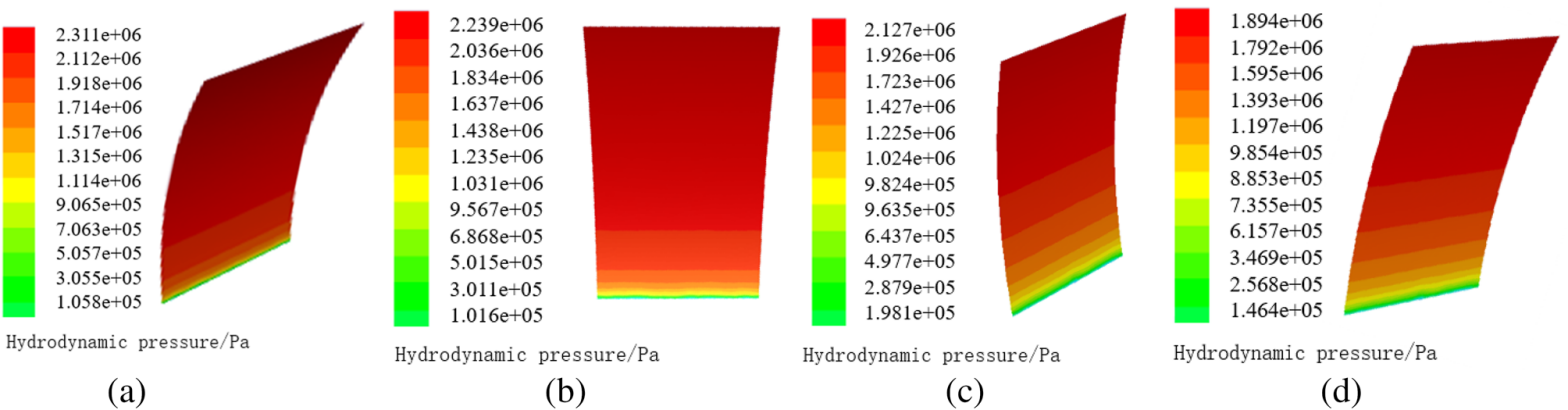
Distribution of hydrodynamic pressure on the surface of radial gate. (**a**) Hydrodynamic pressure distribution at 5 s, (**b**) Hydrodynamic pressure distribution at 10 s, (**c**) Hydrodynamic pressure distribution at 15 s, and (**d**) Hydrodynamic pressure distribution at 20 s.

When maintaining the same opening of the gate, the hydrodynamic pressure distributed on the gate leaf structure may change with the flood discharge time. Figure 5 shows the distribution of the hydrodynamic pressure at 5 s (second), 10 s, 15 s, and 20 s, when the gate opening is 10% as for the finite element model of the gate.

Figure 5 indicates that, when the water flows through the lower edge of the gate leaf, the hydrodynamic pressure of the whole gate leaf changes with the flood discharge time. Moreover, it can be observed that the maximum and minimum values of hydrodynamic pressure decrease gradually at 5 s, 10 s, 15 s, and 20 s, respectively, and the hydrodynamic pressure at the same position also decreases with the increase of flood discharge time. When maintaining the constant local opening height, the hydrodynamic pressure shows a gradually decreasing trend from the top of the gate to the lower edge of the gate. The maximum hydrodynamic pressure appears at the top center of the radial gate, it decreases with the increase of the flood discharge time, and it is 2.311 × 10^6^ Pa, 2.239 × 10^6^ Pa, 2.127 × 10^6^ Pa, and 1.894 × 10^6^ Pa at 5 s, 10 s, 15 s, and 20 s, respectively. While the minimum hydrodynamic pressure is 1.058 × 10^5^ Pa, 1.016 × 10^5^ Pa, 1.981 × 10^5^ Pa, and 1.464 × 10^5^ Pa at 5 s, 10 s, 15 s, and 20 s, respectively.

Once opening the gate, the continuity of water flow forces the water flow in front of the gate to rush to the gate under the action of gravity. The impulse of the flowing water body at the bottom of the gate can produce an instantaneous impact force on the gate, thereby reducing the hydrodynamic pressure near the bottom of the gate. Thus, the hydrodynamic pressure at the bottom decreases as shown in Figs. 5a,b. With the increase of flood discharge time, the influence of instantaneous impact force on hydrodynamic pressure tends to be stable, so the hydrodynamic pressure at the bottom increases as shown in Fig. 5c by comparing with Fig. 5a,b. However, with the continuous increase of flood discharge time, the water level difference between upstream and downstream decreases, and the hydrodynamic pressure at the bottom of the gate begins to decrease, so the hydrodynamic pressure at the bottom is lower as shown in Fig. 5d by comparing with that in Fig. 5c. The hydrodynamic pressure at the top of the radial gate is mainly affected by the water level difference between the upstream and downstream, thus the hydrodynamic pressure reduces gradually with the increase of flood discharge time.

**Figure 6:**
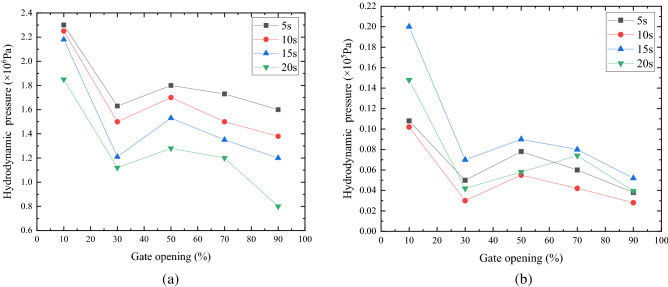
Distribution of hydrodynamic pressure on the surface of radial gate (**a**) Maximum hydrodynamic pressure with different opening, (**b**) Minimum hydrodynamic pressure with different opening.

The influence of gate opening on hydrodynamic pressure is also a problem needing to be considered. In this paper, the hydrodynamic pressure of finite element model structure of radial gate with different opening in the fluid field is calculated. Figure 6 shows the curves of the maximum and minimum values of hydrodynamic pressure at 5 s, 10 s, 15 s, and 20 s and with gate opening of 10%, 30%, 50%, 70%, and 90%, respectively.

As exhibited in Fig. 6, the hydrodynamic pressure generally shows a decreasing trend with the increase of gate opening, while its change is complex and does not linear change with the opening. Moreover, the flow velocity of the water body close to the gate panel increases under the action of gravity with the increase of gate opening. Considering the continuity principle of water body and the conservation principle of mechanical energy, part of the gravity potential energy of water body is temporarily transformed into velocity potential energy, and its velocity vector gradually changes from the orientation of the gate leaf to the bottom of the gate. Accordingly, the hydrodynamic pressure on the gate leaf gradually decreases, and finally disappears.

**Figure 7:**
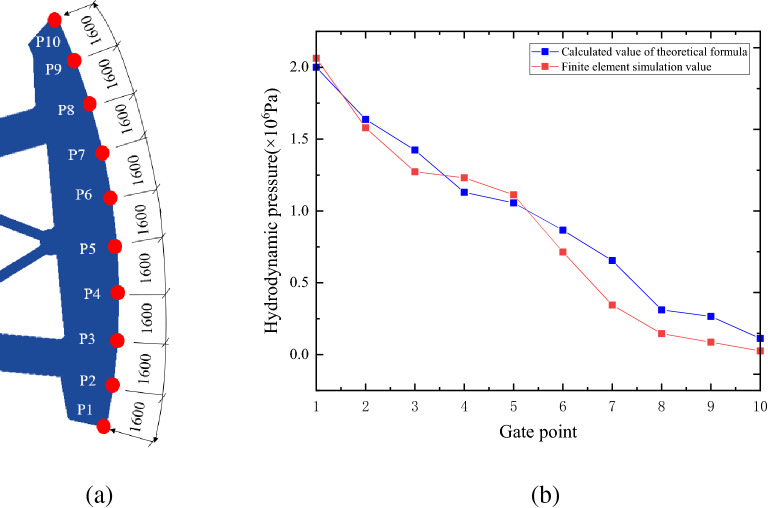
Comparison of theoretical calculation results and finite element simulation results. (**a**) Location map of calculation points, (**b**) Hydrodynamic pressure along the radial gate height.

The hydrodynamic pressure at certain point of the radial gate leaf and at certain time can be calculated by simulation, and it can also be calculated with theoretical formulas (2)– (6) in this paper. Thus, the calculation results with theoretical formula can be used in verifying the correctness of the finite element simulation results in this paper. The center point of the lower edge of the gate leaf is determined as the first theoretical calculation point, and then point is taken every 1.6 m along the height direction of the gate leaf since from the position of the first point, as shown in Fig. 7. The height of the radial gate is 16.4 m in this paper, so total of 10 calculation points are taken when adopting the theoretical formula, with the purpose of verifying the simulation results of hydrodynamic pressure.

On the basis of the fluid structure coupling model shown in Fig. 4, 10 hydrodynamic pressure monitoring points are uniformly set at the center of the radial gate leaf, to investigate the difference between the hydrodynamic pressure values of the 10 monitoring points on the radial gate leaf and the values calculated theoretically, and to further verify the correctness of fluid structure model and finite element simulation of the radial gate. When taking the calculation results of the radial gate model structure at 5 s and with opening of 10% as an example, the comparison curve between the theoretical formula calculation results and the finite element simulation calculation results is as shown in Fig. 7.

Figure 7a presents the monitoring points arranged at the center of the radial gate leaf every 1.6 m, and Fig. 7b exhibits the verification of the theoretical and test values of hydrodynamic pressure on the radial gate at 10 points. By analyzing the results of 10 hydrodynamic pressure points, it is found that the calculation results of the theoretical formula of hydrodynamic pressure coincide well with the finite element simulation results. The total error of 10 groups of comparison data is 8%, which verifies the correctness of the simulation results.

### Nephogram of flow-induced vibration displacement response of radial gate

**Figure 8:**
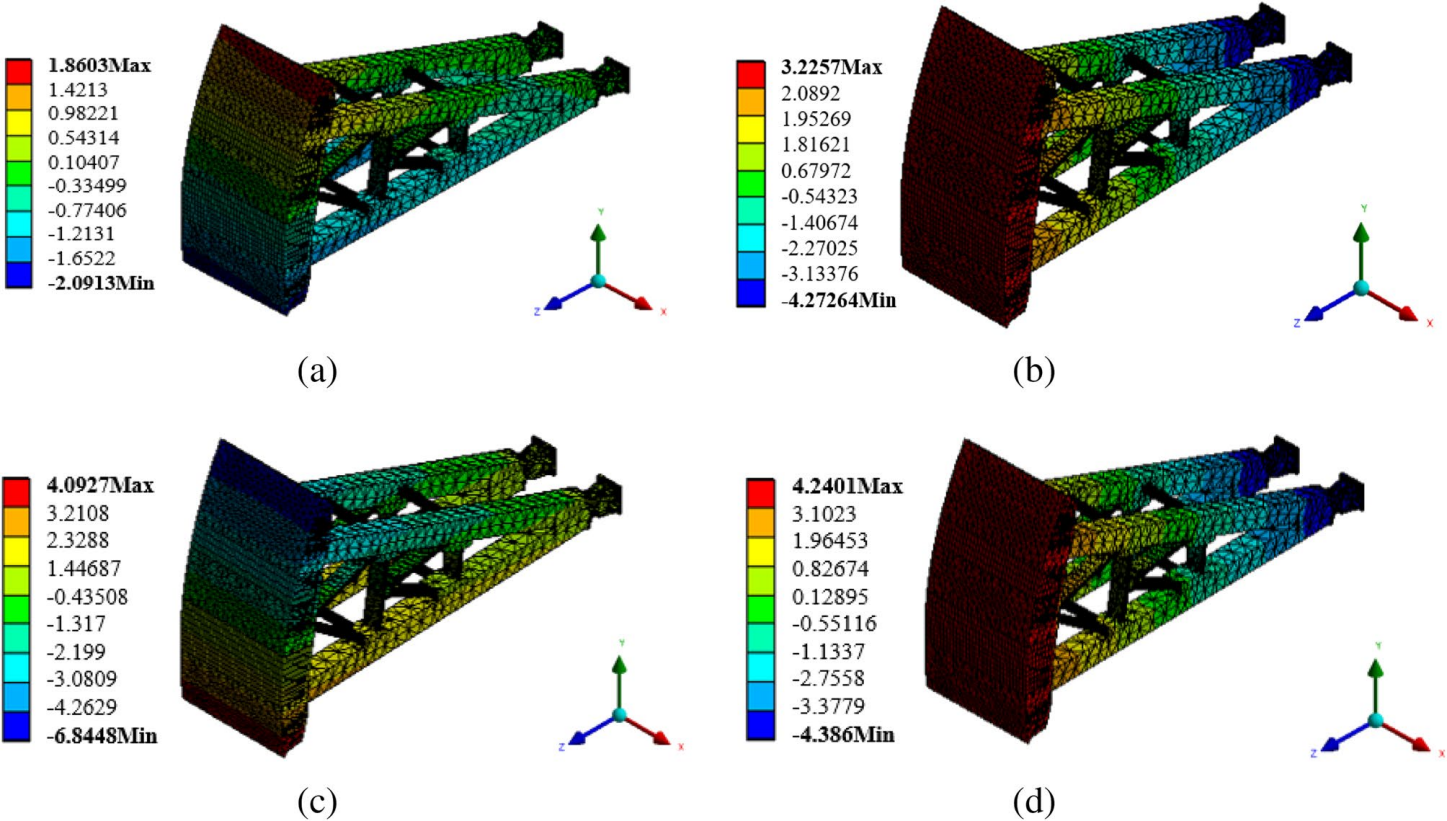
Nephogram of structural displacement response of calculation model. (**a**) Nephogram of displacement response in X direction, (**b**) Nephogram of displacement response in Y direction, (**c**) Nephogram of displacement response in Z direction, and (**d**) Nephogram of total displacement response (unit: mm).

As a water retaining structure, the gate leaf structure of radial gate is strongly vibrated by flow excitation during flood discharge. To obtain the flow-induced vibration characteristics of the radial gate during its operation, the flow excitation time history shown in Fig. 2 is applied to the model structure of the radial gate as a dynamic load, and the acceleration and displacement response of the whole finite element calculation model structure are calculated. When taking the finite element calculation model structure of radial gate with 10% opening as an example, the nephograms of structural displacement responses in X, Y, and Z directions, and the nephogram of total displacement response are as shown in Fig. 8.

The maximum displacement response of the finite element calculation model structure of radial gate under flow excitation, as shown in Fig. 8, is 4.24 mm, when the local opening is 10%. As presented in Fig. 8a, the displacement of the gate is perpendicular to the water flow direction in X direction, and the displacement response component caused by flow excitation is very small. The maximum displacement response at the top of the gate leaf is 1.86 mm, and that at the lower edge of the gate leaf is 2.09 mm, which is opposite to the displacement response direction at the top of the gate. Thus, the gate leaf presents uniform left–right staggered deformation in the plane in X direction, and the maximum relative displacement is about 3.95 mm, which is relatively small compared with the design limit of the radial gate. The displacement response values of the upper arm, lower arm, and connecting parts of the gate structure under flow excitation are also very small in X direction. As exhibited in Fig. 8b, the lower edge of the gate has a positive displacement response in Y direction, with the maximum value of 3.22 mm, and the upper edge of the gate has a negative displacement response, with the maximum value of 4.27 mm. The deformation of the gate leaf is staggered up and down in the plane, and the displacement response values of the upper arm, lower arm, and connecting parts of the gate under flow excitation are distributed in the range of ± 4.0 mm approximately.

It can be observed from Figs. 8c,d that the displacement response of the gate calculation model structure in Z direction is very close to the total displacement response of the gate, that is, the displacement response of the gate mainly occurs in Z direction, and the displacement response of the gate leaf of the gate structure occurs in the water flow direction. The displacement response of the gate leaf is basically caused by the fluctuating water pressure upstream of the water flow, and the flow-induced displacement response of the whole gate leaf is evenly distributed, with the maximum displacement response (4.09 mm) occurring at the center of the lower edge of the gate leaf. The flow excitation makes the gate leaf structure producing a large vibration displacement response as a whole, thereby inducing the vibration of upper support arm, lower support arm, and connecting parts supporting the gate leaf. The displacement response of the support arm structure at the connecting part with the gate leaf is relatively large, and it decreases gradually along the water flow direction. The relative displacement response of the fixed constraint at end of the support arm structure is − 4.38 mm.

In short, under the action of flow excitation, the displacement response of the finite element calculation model structure of the radial gate with 10% opening mainly occurs in the direction along the water flow, presented by the compression deformation in the water flow direction. The displacement response in the plane perpendicular to the water flow direction is small, and the relative deformation caused is generally negligible in the project, when considering that flow excitation in the direction of water flow is the premise of damping control of radial gate in the stages of design, operation, and maintenance.

### Time history analysis of flow-induced vibration displacement response of radial gate

**Figure 9:**
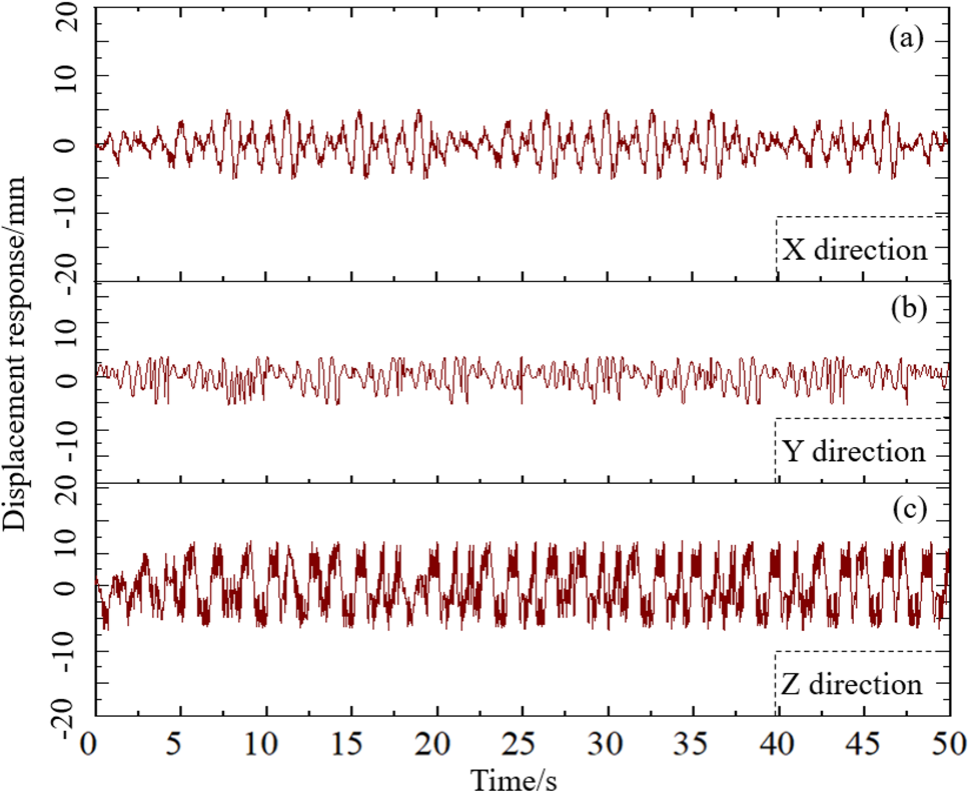
Flow-induced deformation of radial gate.

By giving simulation calculation to the radial gate finite element calculation model structure under flow excitation, the time history of the gate flow-induced vibration response is obtained, which can reflect the variation characteristics of flow-induced vibration response of gate structure with time in the flood discharge stage. The time histories of displacement response in X, Y, and Z directions at the center point of the bottom edge of gate leaf structure are shown in Fig. 9, with 10% structural opening of the finite element calculation model of the radial gate under the fluctuating water pressure.

By analyzing Fig. 9, it can be known that the displacement responses of the finite element calculation model of radial gate show great difference at the structural monitoring points in X, Y, and Z directions under the action of flow excitation, and that the displacement response of the gate structure in X direction is small and tends to increase slightly with the loading time history. From the application simulation of flow excitation load to 50 s, the time history curve of displacement response of the gate structure in e X direction shows two change cycles. The peaks occur in the first 20 s (5.4 s, 10.5 s, 15.3 s, and 19.8 s) of the time history, and the displacement response in the positive X direction reaches the peak values of 5.25 mm, 5.27 mm, 5.26 mm, and 5.25 mm at 5.4 s, 10.5 s, 15.3 s, and 19.8 s, respectively, with the time intervals of 5.1 s, 4.8 s, and 4.5 s, respectively. The variation of time history curve of displacement response between two peaks is basically similar, indicating the regular vibration of the gate structure in the plane perpendicular to the water flow.

Moreover, it can be observed that the displacement response of gate leaf in Y direction is complex as for the finite element calculation model structure of the radial gate. From the application simulation of flow excitation load to 10 s, it appears the peak value (4.1 mm) of forward displacement response in Y direction and the peak value (5.7 mm) of negative displacement response, as well as an asymmetric displacement response in Y direction. The simulation results are related to the change of water level in Y direction. When the flow excitation load is applied to the periods 10–20 s, 20–30 s, 30–40 s, and 40–50 s, the time history curve of displacement response is basically the same as that in 0–10 s, and the peak value of displacement response is roughly same as well, which indicates the certain periodicity of the change of displacement response in Y direction.

As exhibited in Fig. 9, the displacement response of the gate leaf in Z direction is greater than those in X and Y directions as for the finite element calculation model structure of the radial gate, with more complex change. The change of displacement response is basically the same within 50 s after simulation application of flow excitation load. The positive displacement response and negative displacement response show strict symmetry in Z direction. The peak value (7.1 mm) of displacement response may appear many times within the 50 s.

By analyzing the time history curves of the displacement response components of the radial gate finite element calculation model structure in X, Y, and Z directions, it can be known that the point displacement response of the gate structure under flow-induced load is relatively complex, but it also has a certain regularity. Under the action of flow excitation, the peak value of displacement response at each point on the radial gate structure with certain opening appears with certain time interval, with the change trend basically the same in each time interval. The results of simulation calculation are in good agreement with the phenomena observed in the field of radial gate, so they can be used as a reference for analyzing the flow-induced vibration mechanism of radial gate structure.

### Nephogram of flow-induced vibration acceleration response of radial gate

**Figure 10:**
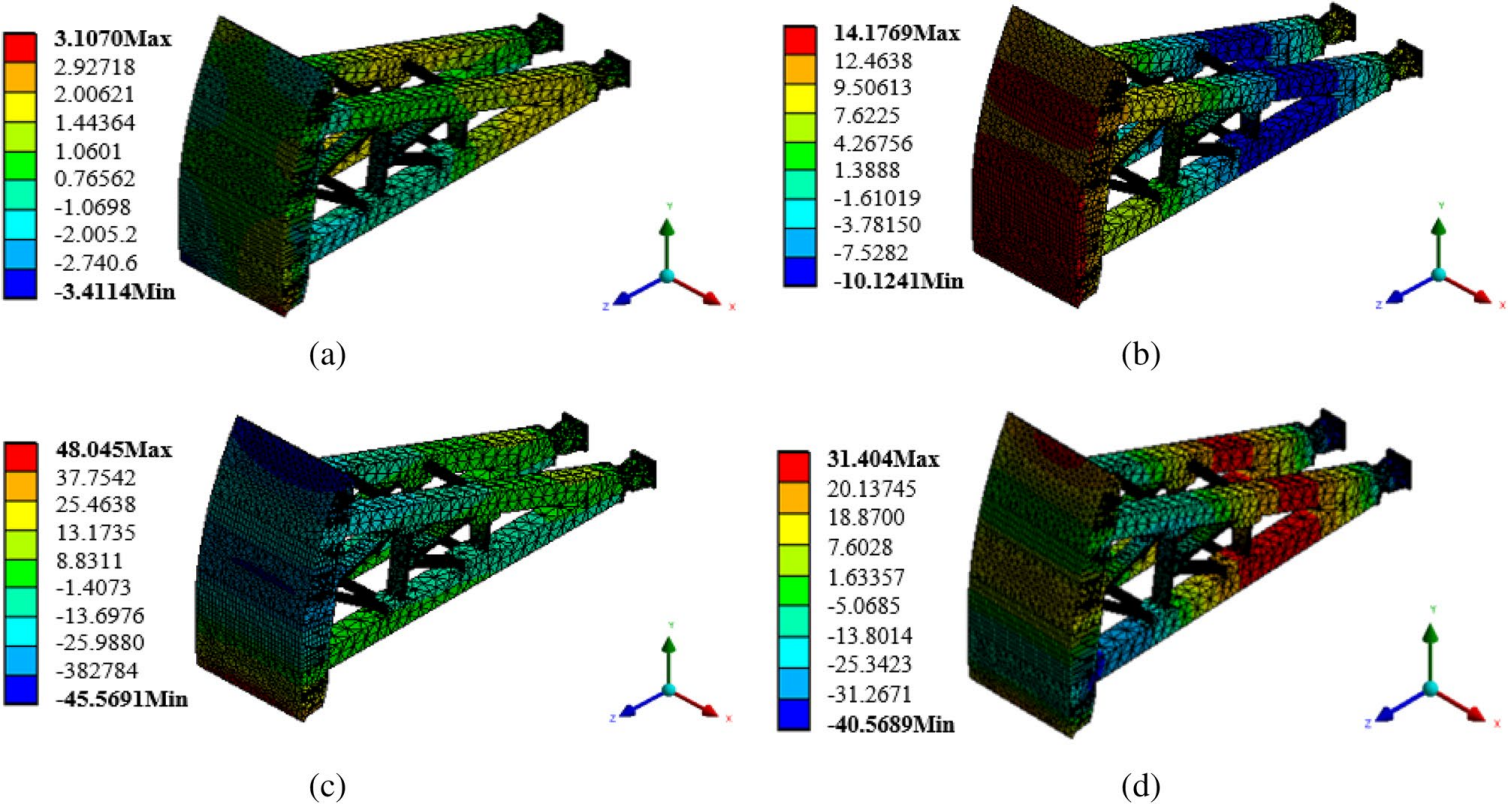
Nephogram of acceleration response of calculation model structure. (**a**) Nephogram of acceleration response in X direction, (**b**) Nephogram of acceleration response in Y direction, (**c**) Nephogram of acceleration response in Z direction, and (**d**) Nephogram of total acceleration response (unit: mm/s^2^).

By applying flow excitation to the gate leaf of the radial gate finite element model structure, the time-domain curve of fluctuating water pressure of the radial gate leaf structure can be obtained. By taking the radial gate finite element model structure with opening 10% as an example, the nephograms of acceleration responses in X, Y, and Z directions, and the nephogram of total acceleration response are as shown in Fig. 10.

It can be observed from Fig. 10 that, under the action of flow excitation, the finite element calculation model structure of radial gate produces acceleration response in X, Y, and Z directions. As presented in Fig. 10a, the relatively uniform acceleration response is generated on the arc surface of the gate leaf of the radial gate in X direction. Moreover, the X direction is perpendicular to the water flow direction, and the acceleration response value in the plane of the gate leaf is small, with the maximum relative acceleration response of 3.10 mm/s^2^. The acceleration response at the connecting parts of the upper arm, the lower arm, and the gate leaf are smaller than that on the arc surface of the gate leaf. Besides, the value of acceleration response of the arm structure gradually decreases along the water flow direction, with the minimum value (0.76 mm/s^2^) showing at the fixed end of the arm. As exhibited in Fig. 10b, the upper and lower support arms give great acceleration responses at 1/3 position away from the fixed end, with the maximum value of 10.12 mm/s^2^. Simultaneously, the acceleration response distribution on the arc surface is quite different, with the maximum value of 14.17 mm/s^2^ and the minimum value of 7.62 mm/s^2^. In addition, the acceleration response on the whole arc surface is uneven, with no trend of gradual increase or decrease. As shown in Fig. 10c, the acceleration response value is larger in Z direction than in X direction. The maximum positive acceleration response (with value of 48.04 mm/s^2^) is distributed at the center of the lower edge of the gate arc surface, and the maximum negative acceleration response (with value of 45.56 mm/s^2^) is distributed at the top of the arc surface^2^. The acceleration response values of the upper arm, the lower arm, and the connecting parts are relatively evenly distributed. The relative acceleration response distribution of the finite element calculation model structure of radial gate with 10% opening, as shown in Fig. 10d, is relatively complex. On the arc surface, the maximum acceleration response is 31.40 mm/s^2^. It is worth noting that there is a large acceleration response at 1/3 position of the upper and lower support arms away from the fixed end.

In short, under the action of flow excitation, the acceleration response of the whole radial gate structure is relatively complex, and that caused by the flow-induced vibration of the gate leaf is large relatively. The effective damping measures should be reasonably considered in design, operation, and maintenance.

### Time history analysis of flow-induced vibration acceleration response of radial gate

**Figure 11:**
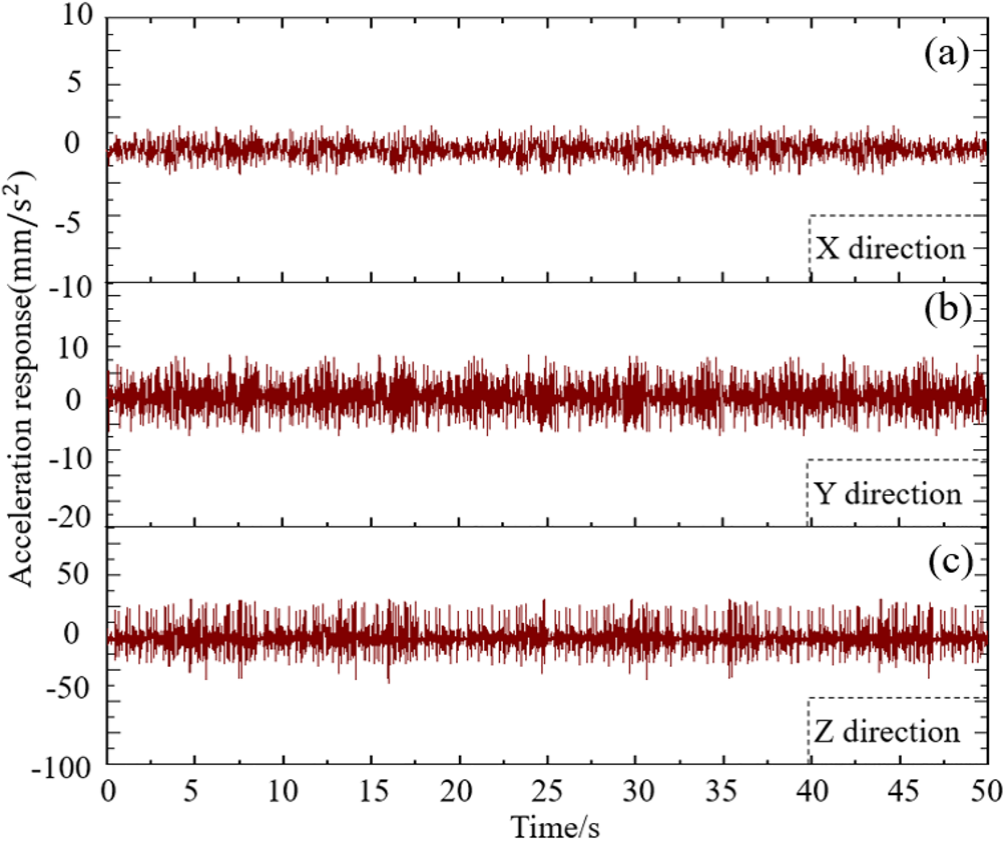
Flow-induced acceleration diagram of radial gate.

By giving simulation calculation to the radial gate finite element calculation model structure under the flow-induced load, the acceleration response time history curve of the radial gate finite element calculation model structure with 10% opening at the center point of the bottom edge in the first 50 s is obtained, as shown in Fig. 11.

By analyzing Fig. 11, the acceleration response of radial gate structure in X, Y, and Z directions under flow excitation can be observed. As indicated in Fig. 11a, the acceleration response change of gate structure in X direction is mainly concentrated between −4 and 4 mm/s^2^ within 20 s, with the peak appearing many times within 50 s. As presented in Fig. 11b, the acceleration response in Y direction is obviously greater than that in X direction, with the range of −15 to 15 mm/s^2^ and the number obviously more in X direction. As exhibited in Fig. 11c, the acceleration response of radial gate structure in Z direction increases obviously by comparing with Fig. 11a,b, with the range of −65 to 65 mm/s^2^ and the peak value of 62.2 mm/s^2^, and there is no obvious law in the peak values of acceleration response.

### Influence of different opening on flow-induced vibration response of radial gate

**Figure 12:**
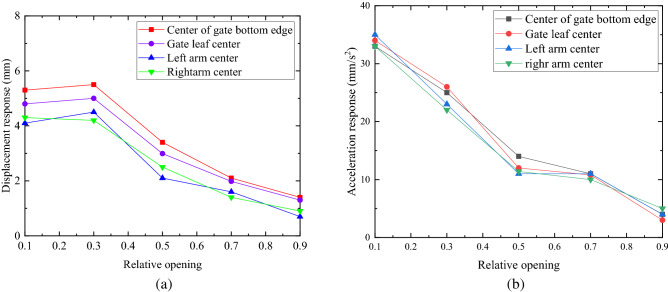
Variation curve of maximum response of gate with relative opening. (**a**) Peak value of displacement response with different opening, and (**b**) Peak value of acceleration response with different opening.

Five groups of flow-induced fluctuating water pressure time histories, with openings of 10%, 50%, 70%, 80%, and 90% as shown in Fig. 2, are input the finite element calculation model structure of radial gate, respectively. The vibration response peak values of radial gate finite element model structures with different openings under flow-induced fluctuating water pressure within 50 s are compared and analyzed. The displacement responses and acceleration responses at the center point of the bottom edge of the gate, the shape center point of the gate leaf, the centroid point of the mid span section of the upper/lower chord of the left arm truss in the water flow direction, and the centroid point of the mid span section of the upper/lower chord of the right arm truss in the water flow direction are selected, with the comparative analysis results of the flow-induced response of the radial gate with different opening as exhibited in Fig. 12.

The peak displacement responses and peak acceleration responses at different positions of the radial gate structure, as shown in Fig. 12, vary with the change of the gate opening. The magnitude of flow-induced fluctuating water pressure, which is input the finite element calculation model structure, directly affects the magnitude of peak displacement response and the peak acceleration response of flow-induced vibration. When the relative opening of gate structure is 10%, 50%, 70%, 80%, and 90%, respectively, the variation trends of displacement response peak and acceleration response peak are basically the same at the same position. As exhibited in Fig. 12a, the maximum displacement response peak (5.7 mm) appears at the center of the gate bottom edge when the relative opening is 10%, while the displacement response peak at the gate leaf shape center is 4.9 mm. Moreover, the displacement response peak at the cross-section shape center of the left and right arm structures is slightly smaller than that of the gate leaf structure. As presented in Fig. 12b, the acceleration response of the radial gate finite element calculation model structure shows the same change trend under the same conditions, and the maximum displacement response peak and acceleration response peak appearing at 50% opening.

In addition, it can be known from Fig. 12 that, considering the displacement response peak and acceleration response peak under the same opening, the flow-induced vibration response peak of the gate leaf structure changes regularly and decreases gradually along the gate height, and the flow-induced vibration response peak at the centroid of the midspan section of the upper/lower chord of the downstream left and right arm trusses is less than that of the gate leaf structure. Meanwhile, the change curve of the response peak value intersects with each other with the change of opening, the change of flow-induced vibration response is relatively complex, and there is no obvious law with the change of the gate opening. Therefore, it is recommended to calculate and control the flow-induced vibration response in design, operation, and maintenance of radial gate to avoid the accumulation of gate damage caused by long-term micro vibration and gate damage.

## Conclusions

With the finite element software, the finite element calculation model structures are established for radial gate hydrodynamics field and solid mechanics field, respectively, and the simulation calculation is carried out. The magnitudes of vibration responses induced by fluctuating water pressure and flow are investigated, and the following conclusions are obtained.

(1) In the radial gate model structure established according to the prototype structure, the fluctuating water pressure on the radial gate leaf can be measured by setting monitoring points. According to the data analysis, the fluctuating water pressure of the gate is roughly − 0.2 to 0.2 kpa, which proves that a better fluctuating water pressure can be obtained in the model test.

(2) The vibration response of hydraulic radial gate under hydrodynamic pressure is different in X, Y, and Z directions, with the maximum displacement and acceleration response occurring in the flow direction. Under the action of displacement response, the gate leaf and arm structure of the gate show deformation in different degrees, and the acceleration change under flow excitation is complex. During flood discharge, the flow-induced vibration of the gate structure is obvious.

(3) Within 50 s before flood discharge, the displacement response of radial gate structure under flow excitation on gate leaf has certain periodic variation characteristics in X, Y, and Z directions, respectively. The peak value of acceleration response of gate structure occurs for many times. The vibration of gate structure under flow excitation has certain periodic characteristics. With the improvement of simulation calculation level, the vibration response, and characteristics of radial gate structure in a longer flood discharge time can be obtained by using this model.

(4) By analyzing flow-induced vibration of finite element model structure of radial gate, it can be observed that the fluctuating water pressure on the arc surface shows a greater dynamic response to the gate blades, upper and lower arms, and connecting parts of the gate, with the obvious dynamic response occurring on the arm structure. In the vibration reduction design, operation, and maintenance, the anti-vibration measures need to be fully considered, to avoid the engineering accidents caused by hydraulic pressure of the hydraulic complex.

## Supplementary Information

Supplementary Information.

## Data Availability

All data generated or analyzed during this study are included in this published article [and its supplementary information files].
